# Large- and Small-Scale Environmental Factors Drive Distributions of Ant Mound Size Across a Latitudinal Gradient

**DOI:** 10.3390/insects11060350

**Published:** 2020-06-04

**Authors:** Orsolya Juhász, Zoltán Bátori, Gema Trigos-Peral, Gábor Lőrinczi, Gábor Módra, Imola Bóni, Péter János Kiss, Dianne Joy Aguilon, Anna Tenyér, István Maák

**Affiliations:** 1 Department of Ecology, University of Szeged, Közép fasor 52, H-6726 Szeged, Hungary; zbatory@gmail.com (Z.B.); lorinczig@gmail.com (G.L.); modragabi@gmail.com (G.M.); imolaboni@gmail.com (I.B.); kisspeterjanos003@gmail.com (P.J.K.); ddaguilon@up.edu.ph (D.J.A.); bikmakk@gmail.com (I.M.); 2 Doctoral School in Biology, Faculty of Science and Informatics, University of Szeged, Közép fasor 52, H-6726 Szeged, Hungary; 3 Museum and Institute of Zoology, Polish Academy of Sciences, Wilcza Street 64, 00-679 Warsaw, Poland; getriral@googlemail.com; 4 Doctoral School of Environmental Sciences, University of Szeged, Rerrich Béla Square 1, H-6720 Szeged, Hungary; 5 Department of Forest Biological Sciences, College of Forestry and Natural Resources, University of the Philippines Los Baños, Laguna 4031, Philippines; 6 Department of Physical Geography and Geoinformatics, University of Szeged, Egyetem Street 2-6, H-6722 Szeged, Hungary; a2na9211@gmail.com

**Keywords:** altitudinal gradient, Bergmann’s rule, *Formica polyctena*, latitudinal gradient, nest mound

## Abstract

Red wood ants are keystone species of forest ecosystems in Europe. Environmental factors and habitat characteristics affect the size of their nest mounds, an important trait being in concordance with a colony’s well-being and impact on its surroundings. In this study, we investigated the effect of large-scale (latitude and altitude) and small-scale environmental factors (e.g., characteristics of the forest) on the size of nest mounds of *Formica polyctena* in Central Europe. We predicted that the change in nest size is in accordance with Bergmann’s rule that states that the body size of endotherm animals increases with the higher latitude and/or altitude. We found that the size of nests increased along the latitudinal gradient in accordance with Bergmann’s rule. The irradiation was the most important factor responsible for the changes in nest size, but temperature and local factors, like the perimeter of the trees and their distance from the nest, were also involved. Considering our results, we can better understand the long-term effects and consequences of the fast-changing environmental factors on this ecologically important group. This knowledge can contribute to the planning of forest management tactics in concordance with the assurance of the long-term survival of red wood ants.

## 1. Introduction

The distribution of species is determined by different environmental gradients, which change along with latitude and altitude [1]. The most important environmental factors having gradients across the globe are temperature, precipitation, and irradiation [1,2]. It is well known that temperature generally decreases and precipitation generally increases linearly with altitude [2], while irradiation decreases with increasing latitude [3]. Ecologists have long-recognised the role of these environmental gradients as ideal laboratories along which to study changes in functional, life-history, and structural characteristics of species [4,5,6,7,8,9,10,11]. As a result, many types of “ecological” rules have been proposed and used by researchers. For instance, according to the well-known ecogeographic pattern (i.e., “Bergmann’s rule”), the body size of endothermic vertebrates increases with decreasing temperature [4].

Bergmann’s rule was firstly applied to a species within a genus [4]. Since then, the relationships between body size, latitude, and altitude have been investigated for several taxa. As a result, studies on endotherms [5,12,13,14] and ectotherms [15,16,17,18] have supported or challenged the validity of Bergmann’s rule [8]. The original explanation behind Bergmann’s rule was related to the heat conservation mechanism, hypothesising that animals with a larger body size can conserve more heat because of their higher surface area to volume ratio [8]. Contrary to endotherms, ectotherms cannot maintain a stable body temperature, therefore, the validity of Bergmann’s rule is questionable in their case. This is the main reason why other hypotheses have also been proposed to explain the above relationships, such as the “starvation resistance hypothesis” and the “resource availability hypothesis” [8]. According to the starvation resistance hypothesis, the individuals with a larger body size (and therefore higher amount of reserves) can endure better the unfavourable periods with low food availability [8]. In sphingid moths, however, the body size shows a latitudinal cline, supporting the resource availability hypothesis [18] that states that the availability of resources (e.g., in time or depending on competition) affects body size, which will increase in areas where resources are available for a longer time and/or the competition is lower [8]. Nonetheless, many ectotherm species follow the Bergmann’s rule, such as *Scathophaga stercoraria* (Diptera), *Psammodromus algirus* (Lacertidae), many bee genera (like Andrena or Halictus), and *Bufo minshanicus* (Bufonidae) [16,19,20,21]. Here, we focus on the changes in the nest size patterns of ants with altitude and latitude in Europe.

According to the superorganism hypotheses, ants can build complex nesting structures that are equivalent to the endotherm organism’s body [6,22,23], and ant workers within these structures can actively regulate the temperature [6,24,25,26,27,28]. Moreover, the size of the nesting structures is in close relation to the colony size (i.e., the increase in any of the three defining parameters of the nest volume will result in a higher number of inhabiting workers) [29], so both the colony size and the size of individual colony members (workers) can be the target of Bergmann’s rule. Indeed, a few studies found that both colony and individual size can increase with increasing latitude [20,30,31,32], whereas altitudinal changes can also lead to alterations in the colony [33] and individual size [34,35]. However, the findings of these latitudinal and altitudinal studies are contradictory and species-specific.

Red wood ants from the *Formica rufa* group can have one of the largest nest structures found in the northern hemisphere [36,37] that correlates with the colony size and is a good indicator of the colony’s well-being [26,38,39,40,41,42]. However, information is scarce about the effects of the latitudinal and altitudinal gradients on these species’ nest sizes that can be related to large- and small-scale environmental factors [41]. Large-scale environmental factors are mostly weather components such as temperature, irradiation, or precipitation that can influence the nest size in many ways. For instance, the daily temperature variance of *Formica* nests decreases with rising latitude [43], but the respiration rate increases with rising altitude [44]. Small-scale environmental factors are mostly related to habitat characteristics, such as the age and distance of the trees around the nests that can influence the size of nests [45,46,47] and can mediate the effects of large-scale environmental factors. For example, *Formica polyctena* builds larger nests in shady forest interiors, whereas *F. rufa* builds smaller nests in well-lit forest edges [36].

According to our knowledge, a comprehensive analysis of large- and small-scale environmental factors affecting the nest size of red wood ants was not in the focus of any study and only a few studies investigated the effects of large-scale environmental gradients on ants within Europe, and among them, the Western European region is over represented [31,32,48]. Red wood ants from the *Formica rufa* group are ideal targets for such a study, as species from this group, like *Formica polyctena*, build large, easily recognisable above-ground nest structures that can be 1.5–3 m wide and up to 2.5 m in height [36]. This complex structure, situated mostly on equatorial-facing slopes (where solar exposure and the incoming solar radiation is higher than in polar-facing slopes) [35], is meant to ensure the thermal stability of the colony and adequate warmth for the larval development in the spring period [37]. Consequently, solar irradiation and temperature can have important effects on the nest size of red wood ants [36,37,49,50,51].

**Figure 1:**
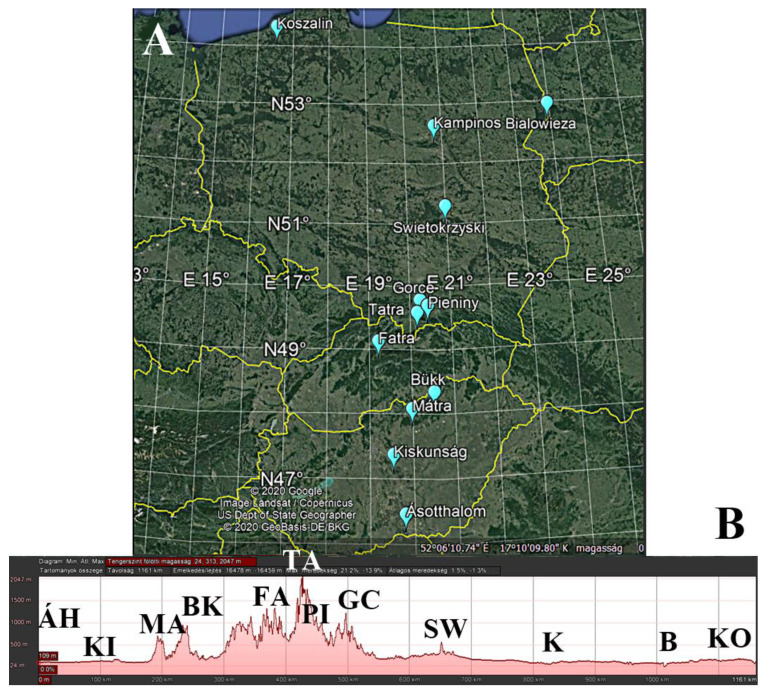
Study regions along the latitudinal (**A**) and altitudinal (**B**) gradient in Central Europe (Hungary, Slovakia, and Poland). Abbreviations: Hungarian lowland: ÁH = Ásotthalom, KI = Kiskunság; Southern Carpathian foothill: MA = Mátra; higher Carpathians (mountain range): BK = Bükk, FA = Fatra, TA = Tatra, PI = Pieniny; Northern Carpathian foothill: GC = Gorce; Polish lowland: SW = Świętokrzyska, K = Kampinos, B = Białowieza, KO = Koszalin.

In this study, we investigated the effects of latitude, altitude (large-scale environmental factors), and forest conditions (small-scale environmental factors) on the nest size of *Formica polyctena* along a south to north transect in Central Europe (Figure 1). We hypothesised that large-scale environmental factors (temperature, precipitation, and irradiation) cause changes in nest size along the gradients and that these changes are in accordance with Bergmann’s rule. Moreover, we also investigated the effects of the small-scale environmental factors (the distance and perimeter of the nearest trees around the nests) that can play an important role in nest size variability. We hypothesised that growing shade (closer, bigger trees) will cause an increase in nest size. 

## 2. Materials and Methods

### 2.1. Study Sites

The study was conducted during the summer months (from the second half of June to the first half of August) of 2017–2019. We sampled the nests of *Formica polyctena* along a 900 km transect (study area hereafter) passing through three Central European countries (Hungary, Slovakia, and Poland). The transect contained a latitudinal (46.215283° N–54.069650° N) and an altitudinal gradient (75–954 m; Figure 1; Table S1), along which comprised the 12 study regions (Figure 1; Table 1). In the Great Hungarian Plain (Hungarian lowland), we sampled two regions (Ásotthalom and Kiskunság), while in the much bigger Polish lowland, we sampled four regions (from the middle of Poland to the Baltic Sea: Świętokrzyska, Kampinos, Białowieza, and Koszalin). To cover the altitudinal gradient between the Hungarian and Polish lowlands, we sampled the foothills of the Carpathians (Mátra Mountains in Hungary and Gorce Mountains in Poland). The study regions in the Carpathians were the Bükk, Fatra, Tatra, and Pieniny Mountains. We chose the number of regions to be representative of the size of the study area, whereas the distance value of the sampling sites is in compliance with their distance from each other (see Statistical analyses).

### 2.2. Sampling Design

In each study region, we sampled the ant nests within 150 × 150 m quadrates (“sampling sites” hereafter), which can be considered a good representation of the territory of *Formica polyctena* [39]. We used GPS (GARMIN Oregon 700 t) to mark the location of nests within the sampling sites. The altitude and latitude of the sampling sites were determined with the help of Google Earth (Google 2019). The sampling sites were managed mixed forests of deciduous and coniferous trees, with coniferous species such as *Picea abies*, *Pinus sylvestris*, *P. nigra*, *Abies alba*, and *Larix decidua*. The most common non-coniferous trees were *Betula pendula*, *Robinia pseudoacacia*, and *Quercus* spp. The age of these forests (based on local forestry databases: NÉBIH, Mapový portal KIMS, Bank Danych o Lasach) was on average 65 years (between 30.75 and 116). The number of sampling sites per region depended on the available *Formica polyctena* populations. In most regions (Ásotthalom, Kiskunság, Bükk, Fátra, Pieniny, Świętokrzyska, Kampinos, Białowieza), we could sample three sites, while in Gorce, Tatra, and Mátra Mountains, we found only two populations, and in Koszalin, only one population. The temperature, precipitation and irradiation data for the regions were obtained from the WorldClim (resolution 30 arc-second, period 1950–2000, Table S1) database [52]. We used whole-year environmental data because despite that red wood ants hibernate during winter, the environmental factors (e.g., temperature) in this period affect early spring survival rates (e.g., raising winter/hibernating temperatures cause elevated death rates in colonies) [42]. The average temperature changed between 10.69 and 4.86 °C from the lowest elevation to the highest, whereas the average irradiation was between 12870.69 and 10055.00 kJ × m^−2^ × day^−1^ and the average monthly precipitation was between 40.17 and 99.5 mm along the transect from south to north (Table S1).

Within each sampling site, we measured the size of each nest (two perpendicular diameters and height). The above-ground nest volume (i.e., semi-ellipsoid) was determined using the following equation:

(1) $$V=\frac{0.75*\pi *r_{1}*r_{2}*h}{2}$$

where *h* is the height of the nest, and *r_1_* and *r_2_* are the two perpendicular nest radii. We used this equation because the above-ground nest volume is closely related to the red wood ant colony size [29,53]. To describe the small-scale habitat characteristics, we also measured the distance of all the closest trees in a circle surrounding the nests relevant to their shading (with laser telemeter SNDWAY SW-T80, accuracy: ±2 mm) and the trunk perimeter of these trees at 1.3 m height, and we also identified the trees at the species level within each sampling site.

### 2.3. Statistical Analyses

We used linear mixed-effects models (LMM, Gaussian error, maximum likelihood fit) to analyse the effect of the latitude, altitude, and forest age on the mean nest size of *F. polyctena* along the transect. In the model, the latitude, altitude, and forest age were included as explanatory variables. The latitude was included as an increasing “Distance value” starting from the southernmost sampling site (Ásotthalom) and added +1 value with every 20 km (in beeline) passing to north (Table 1). The correlation of the background variables (Distance of trees, Perimeter of trees, Temperature, Precipitation, Irradiation) with the latitude (Distance value) was tested with Pearson’s product moment correlation.

We applied a principal component analysis (PCA) on the background variables to test for possible correlations among the large-scale (Temperature, Precipitation, Irradiation) and small-scale environmental factors (Distance of trees, Perimeter of trees) throughout the sampling sites. The PCA was carried out on the covariance matrix based on the average value of the variables. The link between PCA scores (from the first three PCs) of the sampling sites and mean nest size was analysed using linear mixed-effect models (LMMs). The full model included the mean nest size as dependent variables, the PCA scores of the sampling sites for first three PCs as explanatory variables, and the ID of the sampling sites as a random factor.

The effect of the three groups revealed by the PCA (1: Hungarian lowland regions and the Southern Carpathian foothill (Mátra), 2: Mountainous regions and foothills and the Northern Carpathian foothill (Gorce), 3: Polish lowland regions; see also Results) on the mean nest size in the sampling sites was tested with LMM (Gaussian error, maximum likelihood fit). In the model, the mean nest size was included as a dependent variable and group ID as an explanatory factor.

If necessary, the variables were log-transformed prior to the analyses to meet the normality and homogeneity of variances. In all models, the sampling site ID was included as a random factor. Statistical analyses were carried out in R Statistical Environment (R Core Team 2019). LMMs were performed using the *lmer* function from the *lme4* package [54]. Automated model selection was performed with the help of the *dredge* function (*MuMIn* package) [55]. PCA analysis was performed using the *prcomp* function from the *stats* package. The *emmeans* function (*emmeans* package) was used for sequential post-hoc comparisons among factor levels when performing LMM analyses [56].

## 3. Results

Overall, we found 393 nests of *F. polyctena* in our study area. The average nest size for the whole study area was 359.96 (±249.1 SE) dm^3^. The smallest nest size was 0.02 dm^3^ (Kampinos National Park, Poland), whereas the biggest was 3506.9 dm^3^ (Mátra Mountains, Hungary). The smallest average nest size was 80.45 (±54.07 SE) dm^3^ (Kiskunság, Hungary) and the biggest was 908.17 (±575.63 SE) dm^3^ (Białowieza, Poland).

In relation to increasing latitude, the temperature and irradiation decreased from south to north. On the other hand, in relation to increasing altitude, temperature decreased while precipitation increased (see Table S1). We found that increasing latitude caused a significant increase in nest size (LMM *t* = 2.18, *p* = 0.03), whereas the altitude and the age of forests did not (*t* < 0.77, *NS*). We also examined which background variables changed along the latitude, and we found a significant negative correlation with irradiation (Pears. *r* = −0.88, *t* = −10.28, *p* < 0.001) and with temperature (Pears. *r* = −0.41, *t* = −2.42, *p* < 0.05). However, precipitation, the distance of trees from the nest, and the tree perimeter did not correlate with latitude (Pears. *r* < 0.3, *t* < 1.72, *NS*).

**Figure 2:**
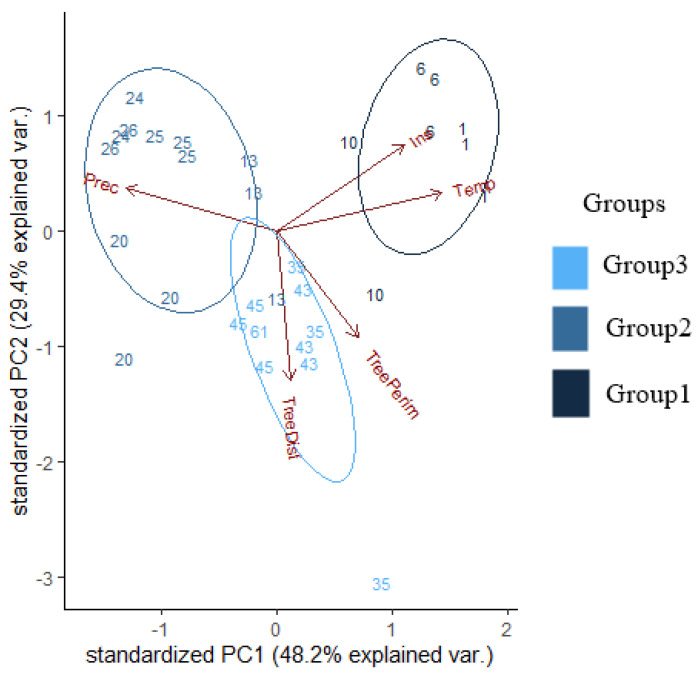
Visual representation of the principal component analyses (PCA) and the three groups (Group1 = Hungarian lowland regions, including the Southern Carpathian foothill (Mátra); Group2 = higher Carpathian Mountainous regions, including the Northern Carpathian foothill (Gorce); Group3 = Polish lowland regions) based on the background variables. Prec = average yearly precipitation, Ins = average yearly irradiation, Temp = average yearly temperature, TreeDist = distance of the closest trees, TreePerim = perimeter of the closest trees.

The PCA analysis of the background variables (Distance of trees, Perimeter of trees, Temperature, Precipitation, Irradiation) revealed that the first axis explains 48.25%, the second 29.37%, and the third 13.15% of the variance in the data (Figure 2; Table 2). The scores of the sampling sites taken according to the first three axes showed that those belonging to PC2 have a significant effect on nest size (*t* = −3.26, *p* < 0.01; Table 2). 

The PCA analyses also revealed three groups (Figure 2): Group1 clustered the sampling sites from the Hungarian lowland regions (Ásotthalom and Kiskunság), Group2 from the mountainous regions (Mátra, Bükk, Fatra, Tatra, Pieniny, and Gorce Mountains), and Group3 from the Polish lowland regions (Świętokrzyska, Kampinos, Białowieza, and Koszalin; Figure 2). We found that the average nest size in Group1 is significantly lower than in Group3 (LMM *t* = −2.3, *p* = 0.02). Group2 did not differ from either of the former two (LMM −1.05 < *t* < 1.43, *NS*).

## 4. Discussions

According to our knowledge, this is the first study about the effects of large- and small-scale environmental factors on the nest size distribution of an ant species. We showed that the nest size of *F. polyctena* increased in Central Europe along a latitudinal gradient with decreasing irradiation and temperature in accordance with Bergmann’s rule. On the other hand, the altitude did not have a significant effect despite the changes in temperature and precipitation along this gradient. Small-scale environmental factors (e.g., tree characteristics around the nests) also played a significant role in the variability of the nest size, mostly in the northern regions. As we expected, more shade (e.g., denser and bigger trees in the nest surroundings) caused an increase in the size of the nests.

Bergmann’s rule predicts that body size increases in relation to decreasing temperature [4], and if we consider the colony of eusocial insects as a superorganism, where the nest structure can be the equivalent of the body [6,22,23], we could apply the Bergmann’s rule by measuring the changes in the size of the nests in relation to latitude or altitude. In red wood ants, not the temperature but rather irradiation seems to have a more pronounced effect on their biology. The lack of the effect of altitude strengthens this finding, as the temperature was the lowest in the mountainous regions. However, irradiation did not decrease significantly towards high elevation. If temperature would be the strongest factor affecting the nest size of red wood ants, we would have experienced an increase in nest size not only with increasing latitude but also with increasing altitude. Moreover, the increases in nest size are in concordance with other findings in ants that explain the larger colony size in the northern regions with the shorter growing seasons than in the temperate latitudes that kill smaller colonies through overwintering starvation [30].

The adequate temperature of nest structures built by red wood ants (e.g., *F. polyctena*) in early spring is achieved by the combined effect of several mechanisms [37]. The nests are usually located in places with high insolation (e.g., equatorial-facing slopes) [36,49], where workers can be often seen sunbathing on the surface of their nest collecting heat from the incoming solar radiation, and the metabolic heat production of the workers helps them to heat up the nests when the temperatures are still low and the snow has not been melted yet [37]. This also emphasises the importance of irradiation over temperature in obtaining earlier the adequate nest warmth. However, the original explanatory mechanism behind Bergmann’s rule may be also valid [8], as the heat conservation of a colony can be better when its surface area to volume ratio is bigger. Consequently, larger colonies can heat up more efficiently their nests (with sunbathing and/or metabolic heat). Besides, the dome shape of the nests (acting like a sun-collector) also helps them to accumulate the incoming solar radiation, and together with the former characteristics, it allows their persistence in areas where irradiation is lower [24,36,37]. Indeed, in our case, irradiation decreased along increasing latitude, leading to an increase in average size towards the northern regions (Polish lowland). This is in line with other studies showing that irradiation (besides temperature) is a strong determinant of the nest occurrence of red wood ants [36,37,49,50,51]. 

The decreased irradiation values found in the Polish lowland regions not only resulted in larger mounds, but also granted higher importance to the small-scale environmental characteristics, such as the distance and perimeter of the nearby trees. In these sampling sites, irradiation is a limiting factor, so the shading (e.g., closer and bigger trees nearby the nests that can have a direct shading effect) and other microclimatic effects of the trees become more important, in contrast with southern regions where the higher amount of irradiation can compensate for the possible negative effects of the close-by trees. A similar correlation between the amount of irradiation and the distance from the closest trees surrounding the nests was observed also in other studies [51,57]. Overall, our study underpins the validity of the Bergmann’s rule in a eusocial species and highlights the importance of large- and small-scale environmental factors that can affect the nest size distribution of these ants.

*Formica polyctena* is one of the most dominant species in ant assemblages of boreal and temperate forest ecosystems, especially of mature coniferous Palaearctic forests (e.g., [58,59]). Moreover, red wood ants are important keystone species and ecosystem engineers [37], and the size of their nests can correlate with their long-time persistence and potential to fulfil their important ecological role [60]. Their presence affects the whole forest ecosystem through their various activities, influencing, among others, the soil composition, the nutrient cycles, and the presence of several other species, from plants to vertebrates [37,61]. Smaller nests, however, can provide a habitat for fewer myrmecophilous species [61], and their impact on forest ecosystems can be also negatively affected, lowering their importance in Central European coniferous and mixed-coniferous forests compared with northern European boreal forests. This knowledge leads to a better understanding of an ecologically important group like red wood ants, especially in the light of the anthropogenic climate change, which causes rising temperatures and sudden changes in weather. This might lead to a further decrease in the nest size of red wood ants [42], but also to the disappearance of their natural habitats [62,63]. Coniferous forests are continuously cut down to prevent bark beetle (Curculionidae: Scolytinae) infestation [63] and clear-cutting also has negative effects on the nest size and vitality of red wood ants [38,42,64]. Therefore, continuous large-scale monitoring of the changes in the nest sizes of red wood ants would be encouraged to be able to detect negative changes and to have time to develop and apply conservation strategies. Meanwhile, we encourage forestry managers to relocate red wood ant nests before clear-cutting to non-disturbed mixed coniferous–deciduous forest stands [65,66]. This, besides protecting the wood ants, through their potential role as biological protective agents, would also protect the forest where they will be relocated [67,68].

## 5. Conclusions

This is the first study concerning the effects of large- and small-scale environmental factors on the nest size distribution of an ant species. Our research showed that the nest size of the red wood ant *F. polyctena* increases across a latitudinal gradient in Central Europe in accordance with Bergmann’s rule. On the other hand, small-scale environmental factors (e.g., the characteristics of trees around the nests) also play a significant role in the variability of nest size, mostly in the northern regions. For example, more shading (e.g., denser, and bigger trees in the nest surroundings) caused an increase in the size of the nests.

Our findings may support forestry and conservation management because the continuous loss of coniferous forests due to bark beetle infestation (Curculionidae: Scolytinae) leads to habitat degradation in a short term. Relocation of the red wood ant nests would help to protect this environmentally important species group and the forest habitats they live in.

## Figures and Tables

**Table 1:** Distance weighted numbers, coordinates, and average altitude of the sampling areas across Central Europe (Hungary, Slovakia, and Poland).

Sampling Site	Distance Value	Latitude (N)	Longitude (E)	Average Altitude (m)
Ásotthalom	1	46.215283°	19.782783°	118
Kiskunság	6	47.121150°	19.504833°	122
Mátra	10	47.827283°	19.974950°	429
Bükk	13	48.081000°	20.502667°	789
Fatra	20	48.882450°	19.211333°	871
Tatra	24	49.325650°	20.153967°	787
Pieniny	25	49.435850°	20.423150°	602
Gorce	26	49.520267°	20.232833°	724
Świętokrzyska	35	50.886933°	21.094567°	302
Kampinos	43	52.361400°	20.792717°	88
Białowieza	45	52.698183°	23.891283°	178
Koszalin	61	54.069650°	16.535000°	90

**Table 2:** The shared percentages of the background variables on the first three PCA axes.

Background Variables	PC1	PC2	PC3
Distance of trees	5.2%	−71%	54.87%
Perimeter of trees	30.11%	−50.69%	−76.3%
Temperature	61.24%	19.9%	−6.58%
Precipitation	−55.56%	20.2%	−28.44%
Irradiation	47.19%	40.72%	17.69%

## Supplementary Materials

The following are available online at https://www.mdpi.com/2075-4450/11/6/350/s1, Table S1: The yearly average temperature (Tavg), precipitation and irradiation of the sampling sites with the number of nests and the average nest sizes (NS), tree distances and perimeters around the nests across Central Europe (Hungary, Slovakia and Poland).

## Research Involving Human and Animal Participants

To obtain the data presented in the manuscript it has been necessary to involve animals, however, during the specimen collection the integrity of the colonies and nest structure were not harmed. This research does not involve human participants.
