# Supplementary material for: Large- and Small-Scale Environmental Factors Drive Distributions of Ant Mound Size Across a Latitudinal Gradient
Source: Insects. 2020 Jun 4;11(6):350. doi: 10.3390/insects11060350 (PMC7348728; doi:10.3390/insects11060350)
Supplement: Supplementary file 1 [file insects-11-00350-s001.pdf]

Table S1. The yearly average temperature (T_{avg}), precipitation and irradiation of the sampling sites with the number of nests and the average nest sizes (NS), tree distances and perimeters around the nests across Central-Europe (Hungary, Slovakia and Poland).

Sampling site	T_{avg} (°C)	Precipitation (mm)	Standard radiation (kJ m^{-2} day^{-1})	Nest number	NS mean (dm^3)	Avr. tree distance (m)	Avr. tree perimeter (cm)
Ásotthalom	10.69	44.72	12870.69	25	325.97	2.62	69.39
Kiskunság	10.47	43.08	12467.03	39	80.45	2.08	51.22
Mátra	8.51	49.25	11812.03	43	696.28	3.97	52.60
Bükk	6.38	57.86	11571.42	52	246.92	3.42	47.85
Fatra	4.86	83.86	10870.36	11	346.31	4.63	40.44
Tatra	5.53	81.38	10645.58	28	231.34	1.66	32.95
Pieniny	6.57	83.11	10752.14	13	486.55	1.60	48.80
Gorce	5.79	95.58	10604.54	18	550.50	1.47	46.85
Świętokrzyska	7.70	49.69	10667.72	63	279.37	3.16	91.85
Kampinos	8.12	40.28	10404.11	27	722.28	11.98	56.02
Białowieza	6.72	50.00	10536.14	13	908.17	4.04	57.69
Koszalin	7.69	56.47	10055.00	79	268.74	3.24	72.81
